# Understanding community perspectives for advancing inclusion of trans and gender‐diverse people in blood donation in Australia

**DOI:** 10.1111/vox.70001

**Published:** 2025-02-19

**Authors:** Rachel Thorpe, Kyle Jensen, Barbara Masser, Peter Bentley, Tarin Dryden, Eleanor Forrest, Andy Kaladelfos, Travis Larcombe, Tanya Pastor, Aaron Saint‐James, Shauna Wilson, Bridget Haire

**Affiliations:** ^1^ Australian Red Cross Lifeblood Melbourne Australia; ^2^ School of Population and Global Health, University of Melbourne Melbourne Australia; ^3^ School of Psychology The University of Queensland Brisbane Australia; ^4^ Faculty of Health and Medical Sciences The University of Western Australia Perth Australia; ^5^ Community Stakeholder; ^6^ Faculty of Law and Justice University of New South Wales Sydney Australia; ^7^ Equality Australia; ^8^ Diversified, School of Education, University of New South Wales Sydney Australia; ^9^ The Kirby Institute University of New South Wales Sydney Australia

**Keywords:** blood donation, blood donors, qualitative research, transgender

## Abstract

**Background and Objectives:**

Trans and gender‐diverse people who wish to donate blood face significant barriers with policies and procedures differing across countries. It remains critical to explore the experiences and perspectives of trans and gender‐diverse people in different locations to understand challenges in local systems. We undertook a qualitative study focussed on procedures and processes affecting trans and gender‐diverse people in Australia, and their views about needed change.

**Materials and Methods:**

Stakeholders collaborated with the researchers to refine the focus of and design the research. Semi‐structured interviews were conducted with 14 trans and gender‐diverse people who were current, past or potential donors. The interviews comprised open‐ended questions about donation experiences, knowledge of current policies and procedures and preferences for change. Interviews were analysed using thematic analysis.

**Results:**

Participants reported varied and inconsistent donation experiences. When compared with current practice, participants preferred a two‐step approach to donor registration that asks for sex reported at birth followed by gender identity. However, they also expressed concern that the two‐step approach could deter new donors and stressed the importance of only collecting information relevant to eligibility assessment. Participants were supportive of a gender‐neutral approach to assess eligibility to donate.

**Conclusion:**

Our study highlights significant barriers and procedural inconsistencies for trans and gender‐diverse individuals when (considering) donating blood. We recommend more inclusive practices including clear communication about data use, rigorous staff training on gender diversity, registration processes that respect all gender identities and adopting a gender‐neutral approach to donor screening.

**Highlights**

- Trans and gender‐diverse individuals face significant barriers and procedural inconsistencies when presenting to donate blood.
- Findings support adopting a gender‐neutral approach to donor screening.
- Blood collection agencies should consider what information about donors it is necessary to collect to ensure donor and recipient safety.

## INTRODUCTION

Blood collection agencies (BCAs) worldwide face calls to ensure that their donation policies and practices are fair and equitable to potential donors who are sexuality and/or gender diverse. This includes ensuring that policy and procedures are based on inclusivity and respect towards potential donors and that temporary or permanent deferrals are aligned with evidence‐based decision‐making.

In recent years, the number of trans, non‐binary and gender‐diverse people presenting to BCAs has increased, including in Australia [1]. ‘Trans’ is an umbrella term for individuals whose gender differs from that reported and recorded at birth, and ‘gender diverse and/or non‐binary’ describes those whose gender identity is fluid and/or who identify outside of binary genders [2, 3]. Although no Australian population‐level studies exist, international research suggests around 0.6% of people identify as trans, and this figure may be higher among younger people [3, 4, 5]. In this paper, we use the term trans and gender diverse to refer to transgender, non‐binary and gender‐diverse people, and we will distinguish between ‘trans’ (people who identify as women or men) and ‘gender‐diverse’ (people who identify as non‐binary) where necessary to reflect differences between these populations.

There has been growing recognition of the barriers faced by trans and gender‐diverse people presenting to donate blood [1, 6]. These challenges occur through the systems and policies of BCAs, as well as through the registration processes and eligibility criteria applied by individual BCAs [1]. The sole BCA in Australia, Australia Red Cross Lifeblood's (Lifeblood) current policy requires donors to provide identification, register with their legal name and identify their gender in binary terms (male/female) using a single field. While this field is used interchangeably by Lifeblood for donor sex or gender, limits in the computer system (ePROGESA) require that either male or female are provided for an answer. This means that a binary trans person can register as their gender, but those identifying as non‐binary or gender diverse are asked to register with their sex reported at birth and potentially not with their preferred name. Requiring donors to register with their legal name can contribute to psychological safety risks and deter donors from returning. Although individuals can change their gender in Lifeblood's online services after registration without official identification, this may not be known to donors. Similar to procedures reported at Canadian Blood Services (CBS), Lifeblood donors may be identified as trans by disclosing to staff, asking to change their gender or name, or from details of their medical history [6]. Lifeblood lacks a specific code to capture trans experience, recording it only in a donor's notes. This information, if missed or not recorded, can result in the donor having to make repeated disclosures. These processes, while not intended to make donation more difficult for trans and gender‐diverse donors, do not conform to key themes identified in a scoping review of providing culturally sensitive care to trans and gender‐diverse individuals. Culturally sensitive care can be improved by implementing procedures, such as inclusive registration tools, that enable early identification of and correct use of pronouns and names [2, 6]. This is an important step in establishing effective communication with trans and gender‐diverse donors [2].

At the time of writing, Lifeblood's donor questionnaire (DQ) includes gender‐specific questions to establish eligibility to donate. Focusing on the previous 3 months, male registered donors are asked about male–male sex while female registered donors are asked about sex with a man who has had male–male sex. In 2018, Lifeblood introduced a supplementary question for donors identified as trans or gender diverse that asks about sexual contact with a male, trans or gender‐diverse partner in the last 3 months. Answering ‘yes’ to any of these questions results in a deferral from donating for up to 3 months.

BCAs use the information entered in the single binary field for sex or gender to inform donor and product safety parameters. For example, Lifeblood applies different normal ranges for donors registered as male and female for estimated total blood volume (TBV) and haemoglobin (Hb) level. These ranges were developed for cisgender donors with trans donors assessed based on their registered gender. However, these parameters can be influenced by gender‐affirming hormonal treatment. Donors taking gender‐affirming hormones have their Hb assessed based on their medically affirmed gender, while those not taking hormones have the Hb range for their sex reported at birth applied. For estimating TBV, Lifeblood applies the cis female predictive formula for all trans and gender‐diverse donors. Further, all donors registered as female, whether cis or trans, and trans men, are excluded from donating blood components associated with transfusion‐related acute lung injury (TRALI) as Lifeblood excludes anyone who could have ever been pregnant. Non‐binary and gender‐diverse donors' TRALI risk is determined on their sex reported at birth.

All BCAs must balance recipient safety, blood sufficiency and the principle of fairness when setting blood donor eligibility criteria, and eligibility criteria must be regularly reviewed to ensure deferrals are supported by the latest scientific evidence [7, 8]. Internationally several BCAs have changed their donor screening procedures from a population‐based approach that defers all sexually active men who have sex with men (MSM) (and many trans and gender‐diverse donors in Australia) to behaviour‐based screening in which all donors are asked the same questions about sexual behaviours [9, 10]. Behaviour‐based screening removes some, but not all, barriers to donation for trans and gender‐diverse people. This approach asks the same screening questions and applies the same criteria to all donors regardless of gender or sexuality. However, options for registration remain binary. Lifeblood is currently considering moving to behaviour‐based screening.

To increase inclusivity, and to acknowledge the growing population demographic who could contribute to blood supplies, understanding the experiences and perspectives of trans and gender‐diverse people regarding blood donation procedures and eligibility is crucial. Given the lack of uniformity in BCA policies and procedures affecting trans and gender‐diverse people, it is vital to gather local data to understand the unique challenges of the local system. This will allow for accurate and relevant recommendations for system improvements and allow their perspectives to be considered, particularly as BCAs change or consider changing sexual behaviour eligibility criteria. Noting the absence of literature focused on the perspectives and experiences of trans and gender‐diverse donors and non‐donors in the Australian context, we undertook a qualitative study. Our focus was on Lifeblood's procedures and processes that affect trans and gender‐diverse people, and their views regarding change to enhance inclusivity and fairness in blood donation practices.

## MATERIALS AND METHODS

Ethical approval was obtained from Lifeblood's human research ethics committee (2022#38). We aimed to design research that was focussed on the issues of concern for trans and gender‐diverse people presenting to donate blood and inform Lifeblood policies. Accordingly, the project leaders worked with a stakeholder group of seven trans and gender community representatives and four Lifeblood staff employed either in donor‐facing roles or in designing and monitoring blood donation safety criteria to co‐design the research [11]. Stage 1 involved three meetings working with this group to gain their perspectives on donation policies, procedures, and experiences and to inform an interview study. Stage 2 involved conducting semi‐structured interviews (November–December 2022). After the interviews, the stakeholder group reviewed the findings and developed key recommendations.

### Recruitment and participants

As Lifeblood cannot systematically identify trans and gender‐diverse donors, interview participants were recruited via social media advertisements disseminated by the researchers and stakeholders. The advertisement explained the purpose and scope of the study and encouraged participation from trans and gender‐diverse people from diverse ethnic backgrounds and Aboriginal and Torres Strait Islander people. Participants were eligible if they were trans, gender‐diverse or non‐binary and either current or past blood donors or non‐donors who had looked at the eligibility criteria, interacted with the Lifeblood call centre, visited a donor centre or considered donating but chose not to.

Those eligible and interested scanned a QR code to complete a short screening survey to provide demographic and contact details and indicate a preferred interview time.

Thirty‐four people submitted the survey, and interviews were completed with 14 people. The remaining people could not be contacted to schedule an interview. All interviewees provided verbal consent.

### Interview procedure

Interviews were semi‐structured and employed open‐ended questions with prompts to encourage conversation between the interviewer and participant. The participants were able to expand beyond the specific questions asked. Participants were able to review their transcripts. The interview topics included experiences of donating blood or of attempting to donate, knowledge of Lifeblood's policies affecting trans and gender‐diverse people and suggestions for improving the inclusion of trans and gender‐diverse people in blood donation. After clarifying that currently in Australia population‐based deferral criteria are applied to sexually active MSM and trans and gender‐diverse donors who have sex with men or trans and gender‐diverse people, the interviewers asked participants specific questions about this approach and their views regarding change, with explanations of the behaviour‐based screening approach adopted in England given as an example of an alternative approach [12, 13].

Interviews were conducted by one of two interviewers and ranged from 25 to 59 min. All interviews were audio‐recorded and transcribed verbatim, with transcripts verified against recordings. Participants were offered a 100 AUD gift card as reimbursement for participation.

### Analysis

One researcher thoroughly read each transcript before coding the interviews by identifying and labelling thematic codes relating to donation barriers, motivators and views on donation policies, and donation procedures. These initial codes were discussed and refined through discussion within the project team. Higher level themes were derived from reviewing the initial codes and identifying overarching themes linking them [14].

The data are presented in order of the donation process and the themes and sub‐themes within these topics.

## RESULTS

### Participants

Participants' demographic characteristics are shown in Table 1. Six were current donors, five were former donors and three had not donated in Australia but were interested in donating.

**TABLE 1:** Participant characteristics.

Age (mean, range)	30 (24–44)
Gender (*n*)^a^	
Non‐binary and transmasculine	4
Trans man/transmasculine	3
Non‐binary	3
Transgender woman/transgender female	2
Gender neutral	1
Transgender and transmasculine	1
Donation experience (mean, range)	29 (1–105)

^a^ Participants provided their gender in an open‐text response with multiple responses possible.

Seven current or former donors started donating during high school or university, while the remainder started to donate more recently. Two non‐donors had been ineligible until recently because of Australia's former variant Creutzfeldt–Jakob disease (vCJD) deferral, while one had donated overseas.

### Registering to donate and completing the DQ

#### Experiences of registration are variable and inconsistent

The experiences reported by current and former donors were varied and inconsistent, both within and between individuals.

Most trans donors reported being able to register with their gender, while three non‐binary participants highlighted the current binary registration options as a barrier because they had to identify as male or female. Despite most being able to register with their gender, participants identified how using the ‘gender’ field to record either sex or gender caused difficulties at later stages of the donation procedure. For example, one non‐binary participant had registered as male, which differed from their sex reported at birth, but was unsure whether staff knew this as there was no clear procedure to capture the relevant information. This donor was therefore unsure whether staff applied the correct criteria related to Hb, product use and TBV to their donations.

Participants who had affirmed their gender since registering to donate but not changed their name or gender with Lifeblood reported that staff referring to them with their incorrect name, title and pronouns, or using gendered language based on how the donor presented. Participants reporting these experiences understood these terms were used with good intent. Other participants reported that staff had found a way to record their preferred pronouns.

Non‐donors and some former donors expressed anxiety about navigating the binary gender options, identifying these options as a barrier to donating for themselves and other trans and gender‐diverse people. Non‐donors were also anxious about being treated differently from other donors while registering. One past donor, who no longer identified in the gender they were registered under, noted that this was a barrier to returning to donate.

When asked about their views on the donor registration process, most participants indicated that a two‐step approach that asks for sex reported at birth followed by a question about gender identity would be more acceptable than the current system. Two non‐donor participants did not indicate whether asking about sex reported at birth was acceptable. All participants preferred that gender identity options were expanded to at least include ‘non‐binary’ and ‘I/they use another term’. Drawing on experiences in other healthcare settings, participants reported that if sex reported at birth was needed and this was explained to donors, they would accept being asked this.

I come across this a lot of the time and within the health system there are so many situations where biological sex or sex assigned at birth is really pertinent, like looking at pathology ranges or anything like that. So, I understand and totally accept that Lifeblood Red Cross need to capture that in the initial stages (P 2)

However, participants thought that asking all prospective donors their sex reported at birth may deter other trans and gender‐diverse people from becoming donors.

We asked participants about their experience of completing the DQ and about any views regarding change. While participants in general found the DQ questions acceptable, a small number reported that all men should also be asked whether they have been pregnant since their last donation, as the current question response options of yes/no/I am male does not take into account that trans men could be pregnant. They noted that donating could be harmful to trans men if they have recently been pregnant.

There's this question about pregnancy. Are you pregnant? And when I've done it, the answers were “Yes, No, I am a man.” And I hate that, because men can be pregnant, and I don't see how it would offend men to just say no to that question. That just seems a bit non‐inclusive (P 7)

#### Donation and disclosing gender identity

Donor participants reported being unsure if, and what, information about their trans or gender‐diverse experience was recorded in their donor notes. Some described gender identity being raised by staff at the interview stage either if they had listed hormone therapy when detailing their current medications or reported recent gender‐affirming surgery. Other participants had told staff that they were trans or gender diverse when calling to ask questions about eligibility to donate. Those who had disclosed this information were comfortable doing so; however, they considered other trans and gender‐diverse people may not want to have to do this to donate. Some participants said that their gender identity had not come up during the donation process, and others did not want to bring gender identity into blood donation. Participants noted that donors will not be identified by the BCA as trans and gender diverse if they are not taking hormones or have not recently had surgery that is identified by staff as related to gender affirmation, unless they volunteer this information.

### Donor interview

#### Interview experiences are inconsistent

Participants reported that their experiences during the donor interview, in which a staff member reviews the donor's responses to the DQ and undertakes a basic medical assessment, varied depending upon the individual staff member involved, the donor centre and whether the donor had been identified as trans or gender diverse. Participants reported that some staff appeared unsure of how to identify the correct Hb range, calculate TBV or how to apply sexual behaviour deferral criteria to them. Participants who mentioned these experiences reported that their interview was very long, interrupted by staff checking procedures with a supervisor, and staff asking the donor themselves how to apply the criteria.

The things that I have experienced whilst donating blood are things like the interview staff are often really confused about, “Do I check for the male or the female levels (of) haemoglobin? Do I take the male or female levels of whatever,” those sorts of things. Sometimes they ask me (P 8)

Participants also reported that the supplementary question regarding sexual activity with a trans or gender‐diverse person was inconsistently asked. As they were not always asked this question, participants were unsure whether this was the correct procedure or whether they were being discriminated against. Accordingly, some recalled being offended when the question was asked.

Participants reported mixed views of the acceptability of these experiences. For example, one said that staff had provided clear explanations of why different ranges were used for Hb and TBV, which helped with the participant's own understanding and acceptability. Others reported that staff were not able to explain procedures, and expressed concerns that procedures were not working well to protect recipients.

Several participants reported that staff sometimes asked them to play ‘educator’ about being trans or gender diverse—either by mentioning they had not interviewed a trans donor before or asking them personal questions unrelated to blood donation. Most participants said that staff may have good intentions when entering into these conversations, such as making small talk, and one participant found it positively that a staff member wanted to learn about the most appropriate way to assess and interact with gender‐diverse donors.

So, it was nice to know that they were looking at my donation as an educational experience for them, and they could use that to then maybe change the language or change the way they may approach other people who might come after me, who are presenting differently as well (P 6)

However, most described these conversations as very stressful as they just wanted to donate blood. Participants thought that these conversations could bring up traumatic experiences for some trans and gender‐diverse donors.

#### Understanding of policies and procedures affecting trans and gender‐diverse donors

Participants had mixed understandings of how blood donation parameters were calculated, about TRALI risk, and why staff had difficulty with these parameters during the interview. Some participants reported learning that the criteria were applied differently based on pregnancy history and hormone use. However, participants' understandings were often incorrect. Participants expressed interest in learning how the BCA currently assesses these donation parameters and preferred this information to be explained to them upfront, which they felt would greatly enhance transparency and build trust.

#### Understanding of eligibility criteria

Participants' understanding of eligibility criteria affecting trans and gender‐diverse people varied usually based on their own experiences, such as of being asked the supplementary question or not, and of deferral. Current donor participants who had been asked the supplementary question inconsistently were unsure of the correct procedure. Participants also provided examples of operational difficulties and inconsistencies relating to understanding and applying eligibility criteria. For example, one donor who was non‐binary but registered as male and taking testosterone questioned whether their partner would be eligible to donate. Another noted that after they affirmed their gender, they were no longer eligible to donate because they were classified as male and as having male–male sex despite being in the same monogamous relationship as prior to the gender affirmation. Another participant noted that while they were deferred because they were identified as trans due to hormone use, their non‐binary partner could donate because they were not identified as non‐binary by staff and not asked the supplementary question.

Current donor and some past donor participants who had not been deferred reported being unsure of the eligibility criteria affecting trans and gender‐diverse people. For example, one current donor thought that these criteria only applied to men who had sex with men and therefore did not affect them.

### Perspectives on eligibility criteria

#### Eligibility should be based on behaviour rather than identity

All participants thought that eligibility to donate should be based on sexual behaviour rather than gender identity. While understanding the need to minimize risk to recipients, participants found the current population‐based approach discriminatory, too broad and inaccurate because it categorized trans and gender‐diverse people as higher risk than cisgender people regardless of their specific sexual practices. They thought this approach unnecessarily prevented trans and gender‐diverse people from donating and questioned why the BCA could not differentiate between high and low‐risk groups within the diverse identities and sexual practices of trans and gender‐diverse people.

I understand that kind of broad categories are used because at a kind of population level or at a demographic level, trans people and men who have sex with men are more likely to have HIV, but I feel like a more accurate question that would capture that risk would be related to, “Have you had a new sexual partner in the past deferral period?” (P 9)

For these reasons, all participants were supportive of Lifeblood adopting a behaviour‐based approach to assess eligibility to donate, as has occurred overseas [9]. Further, they thought that asking everyone the same questions would avoid gender identity becoming a focus of the donor interview.

Participants also said that greater equity in donor screening would be well received within the trans and gender‐diverse communities. Most reported that they would be able to donate if assessed using a behaviour‐based approach, with few barriers identified.

## DISCUSSION

BCAs must be as inclusive as possible to maximize the number of people who wish to donate, avoid unnecessary deferrals and ensure the psychological and physical safety of trans and gender‐diverse donors and staff. In this paper, we discussed the experiences and perspectives of trans and gender‐diverse donors and non‐donors in Australia—a setting not previously examined, but where policies are similar to those applied by other BCAs [1]. In drawing attention to the donation processes and criteria that affect trans and gender‐diverse people, practices in Australia are inconsistent and often not optimal. Our findings and recommendations will be of use to organizations looking to be more inclusive.

Some of the barriers reported relate to assumptions about gender and sexuality that have historically been embedded in blood donation and other healthcare systems. For example, binary gender options for donor registration limit participation of non‐binary and gender‐diverse people as well as limiting the capture of accurate information about the genders of those who do. Additionally, cisnormativity, the assumption that a donor's sex and gender are the same and have not changed, leads to processes not designed to capture accurate information about trans and gender‐diverse people who present to donate. This results in insufficient attention to correct pronoun usage, names and titles. Incorrect usage may result in donors feeling unsafe, disrespected and undervalued. Inaccurate recording of a donor's gender experiences may also impact the accuracy of calculations around TBV and other parameters designed to protect the safety of donors and recipients. As our data demonstrate without a mechanism to systematically record trans and gender‐diverse donors in the donor database, there is a risk that current procedures may not be applied correctly, and those who want to donate will have suboptimal and inconsistent experiences. Further research is needed to examine the best way to apply donor safety measures to non‐binary donors [15]. Our findings also demonstrate that applying sexual behaviour screening questions based on gender identity, such as the supplementary question asked of trans and gender‐diverse donors, is viewed as discriminatory and not evidence‐based.

In addition to moving away from a population‐based approach to sexual behaviour screening, one way to address identified problems relating to assessment of donation parameters is to capture information about both sex and gender at registration through a two‐step question, and to broaden the options for gender. Pandey et al. [1] report that a working group focused on the software currently used by Lifeblood and other BCAs to capture information about sex/gender is working to make the two‐step approach possible. A Canadian study exploring two‐spirit, trans, non‐binary and other gender‐diverse blood and plasma donors' perspectives for asking about sex and gender recommended not introducing a two‐step approach unless this information is needed for donor and/or recipient safety [15]. In contrast, we identified broad agreement with the two‐step approach when it was compared with current practice. Yet we also note concerns from participants that some will not want to disclose their gender identity when donating blood, do not want their gender identity to be a focus when donating and that asking a two‐step question and recording information about sex reported at birth and gender identity may deter non‐donors from donating. This indicates that additional research is required to explore the issue further. Following this, and in agreement with Haw et al., we believe it is important for BCAs, including Lifeblood, to consider what information about donors is necessary to ensure donor and recipient safety, why it is needed, and what alternatives may be preferred. Further, the rationale for collecting information about sex and gender needs to be explained clearly to donors and those considering donation, including how that information will be stored and who can access it. Our findings suggest that education would be required for staff, donors and non‐donors if these changes were brought in. That is, while our participants had a good understanding of the differential meanings of sex and gender, it was suggested that donor centre staff and the broader donor population may not understand the difference between these terms or why this information is relevant to blood donation.

Our data demonstrate that it is a priority that donors and the public can access accurate information about blood donation policies and procedures relevant to them. Providing clear and transparent information will help to build trust between the BCA and trans and gender‐diverse donors and communities and avoid situations in which people are unsure why certain processes are being followed or feel discriminated against. Staff training is an important aspect of this. Further, eligibility criteria, and appropriately designed procedures, including automated eligibility tools relating to trans and gender‐diverse donors, should be able to be easily understood and applied by staff working in donor‐facing roles.

Our study is limited due to the small sample size and convenience sampling. Further, to our knowledge, no other country includes a question about sex with a trans and/or gender‐diverse person in donor screening. However, we believe our findings will be of use to other BCAs that use ePROGESA, only offer binary options for donor registration, and where a population‐based approach to donor screening is used.

Our study highlights a strong commitment to blood donation among trans and gender‐diverse individuals that is underscored by significant barriers and procedural inconsistencies faced by members of these communities. Supporting investment in changes, both procedural (e.g., at minimum including a non‐binary option) and in practice (through staff training), will ensure that trans and gender‐diverse donors are recognized as a valued population in the donor pool. Adopting more inclusive practices, informed by our data, would involve clear communication about data use, rigorous staff training on gender diversity, and a registration process that respects all gender identities.

Embracing these changes would not only improve the donation experience for trans and gender‐diverse individuals but also contribute to a more equitable health system. This approach aligns with the broader international trend towards inclusivity in blood donation practices and underscores the importance of adapting policies to meet diverse donor needs.

## CONFLICT OF INTEREST STATEMENT

The authors declare no conflicts of interest.

## Data Availability

The data are not available from the author due to anonymity and confidentiality of participants.
